# The Value of Non-Instrumental Information in Anxiety: Insights from a Resource-Rational Model of Planning

**DOI:** 10.5334/cpsy.124

**Published:** 2025-02-12

**Authors:** Bilal A. Bari, Samuel J. Gershman

**Affiliations:** 1 Department of Psychiatry, Massachusetts General Hospital, Boston, MA, USA; 2 McLean Hospital, Harvard Medical School, Belmont, MA, USA; 3 Department of Psychology and Center for Brain Science, Harvard University, Cambridge, MA, USA; 4 Center for Brains, Minds, and Machines, Massachusetts Institute of Technology, Cambridge, MA, USA

**Keywords:** anxiety, resource rationality, decision making, uncertainty

## Abstract

Anxiety is intimately related to the desire for information and, under some accounts, thought to arise from the intolerance of uncertainty. Here, we seek to test this hypothesis by studying the relationship between trait anxiety and the willingness to pay for non-instrumental information (i.e., information that reveals whether an event will happen but cannot be used to change the outcome). We model behavior with a resource-rational model of planning, according to which non-instrumental information is useful for planning ahead, but paying for this information only makes sense if the anticipated benefits of planning outweigh the cognitive and financial costs. We find a bidirectional effect of trait anxiety factors on information seeking behavior: those with high trait somatic anxiety exhibit a stronger preference for non-instrumental information, whereas those with high trait negative affect exhibit a weaker preference. By fitting the resource-rational model, we find that this divergent desire for information arises from the utility of obtaining information for future planning (increased in somatic anxiety, decreased in negative affect). Our findings lend support to the intolerance of uncertainty hypothesis in somatic anxiety and highlight the importance of studying anxiety as a multifactorial construct.

## Introduction

Anxiety has been conceptualized as an ‘epistemic’ emotion, intimately related to the pursuit of information (Miceli and Castelfranchi, 2005). Anxiety increases in response to uncertainty, and its aversive nature motivates the resolution of uncertainty (Caplin and Leahy 2001; though not all forms of uncertainty are associated with displeasure, see Loewenstein 1987). When anxiety becomes pathological, its effects can generalize across domains, such that seemingly innocuous circumstances give rise to intolerable distress (Szuhany and Simon, 2022). These baseline levels of anxiety define trait anxiety (i.e., stable, enduring anxiety irrespective of contextual factors), which vary across individuals and will become the focus of this paper. One influential perspective states that pathological trait anxiety arises from the intolerance of uncertainty (Dugas et al., 1998, 2001; Carleton et al., 2007, 2012).

In this paper, we explore the epistemic view of trait anxiety through the lens of non-instrumental information preferences (Kreps and Porteus 1978; Wu 1999; see also Bennett et al. 2020). These preferences appear in scenarios where a person can gather information about a future outcome but cannot change the outcome (e.g., watching a tense sports game, anticipating the outcome of a job interview, or awaiting the results of a diagnostic medical test). At first glance, learning about an outcome in these scenarios can only reduce uncertainty (although we will shortly argue that there is more going on). It seems irrational to want to know whether an outcome will happen in these scenarios, yet people act on these desires, frequently choosing to pay money (Pierson and Goodman, 2014; Jiwa et al., 2021), sacrifice a proportion of future earnings (Bennett et al., 2016, 2020; Brydevall et al., 2018), expend physical effort (Goh et al., 2021), and even endure pain (Bode et al., 2023) to gain non-instrumental information. These preferences are typically amplified by the degree of uncertainty reduction (Charpentier et al., 2018; Van Lieshout et al., 2018; Kobayashi et al., 2019; Sharot and Sunstein, 2020). Why do people value non-instrumental information? Note that some of these studies used hypothetical choices without feedback (like ours), whereas others used choices with real outcomes, a point which we return to in the Discussion.

Pierson and Goodman (2014) proposed that non-instrumental information preferences arise from the utility of planning ahead: even if information doesn’t affect the outcome itself, it can affect what you will do with the outcome. For example, learning about the outcome of a job interview earlier provides more time to think about what projects to take on in the new job. According to Pierson and Goodman’s model, such information is treated as if it was instrumental. Importantly, the value of information depends both on the expected utility of planning ahead and the disutility of devoting limited cognitive resources to planning. The “resource-rational” decision balances these factors to optimize expected utility (Lieder and Griffiths, 2020; Bhui et al., 2021). Consistent with the model predictions, Pierson and Goodman showed that people generally have a stronger desire for information when the probability of the outcome is higher, except for a slight reduction in desire when the probability of the outcome is very high and the information is costly. The intuition for this result is that the utility of planning is higher when the outcome is more likely to occur (if the outcome is not going to occur, there’s no point planning for it). When information is costly, people may be deterred from paying to learn about outcomes that are highly likely to occur anyway; the expected utility of planning doesn’t outweigh the cost of information.

In this paper, we use the experimental task and resource-rational model of Pierson and Goodman to better understand decision making in trait anxiety. The model offers one way of formalizing an intolerance of uncertainty account, according to which high trait anxiety promotes stronger desire for information. In the model, this could arise through several possible pathways: higher utility for the outcome, lower cost of planning, lower cost of information, lower utility of leisure, or weaker loss aversion. We assess the evidence for each of these pathways by analyzing parameter estimates.

Importantly, recent work has argued that anxiety is not a unitary construct, and that different anxiety factors influence behavior in distinct ways (Wise and Dolan, 2020; Fan et al., 2023; Witte et al., 2024). This is consistent with the multifactorial nature of anxiety, which can manifest with a number of psychological (e.g., feelings of apprehension, impaired concentration, irritability) and physical (e.g., elevated heart rate, rapid breathing, and tremulousness) symptoms. We build upon previous work (Fan et al., 2023) and decompose trait anxiety into four distinct components: somatic anxiety, cognitive anxiety, negative affect, and low self-esteem. This allows us to develop a more fine-grained and clinically meaningful picture of the relationship between trait anxiety and information preferences.

## Methods

### Subjects

We first recruited 80 subjects (the ‘discovery’ sample; mean age ± SD, 42.9 years ± 12.8, range 23 to 73; 54 male, 25 female, 1 non-binary) from Amazon Mechanical Turk and hosted the experiment on Qualtrics. On the basis of the discovery sample, we pre-registered all our analyses and models at https://aspredicted.org/N49_GBQ and collected data from *N* = 227 subjects (the ‘pre-registered’ sample; mean age ± SD, 40.7 years ± 10.7, range 21 to 77; 138 male, 87 female, 1 non-binary, 1 prefer not to say). In this manuscript, we report our pre-registered analyses in the pre-registered sample in the main text and results from the discovery sample in the supplement. All subjects gave informed consent and the Harvard University Committee on the Use of Human Subjects approved the experiment.

### Task

**Figure 1:**
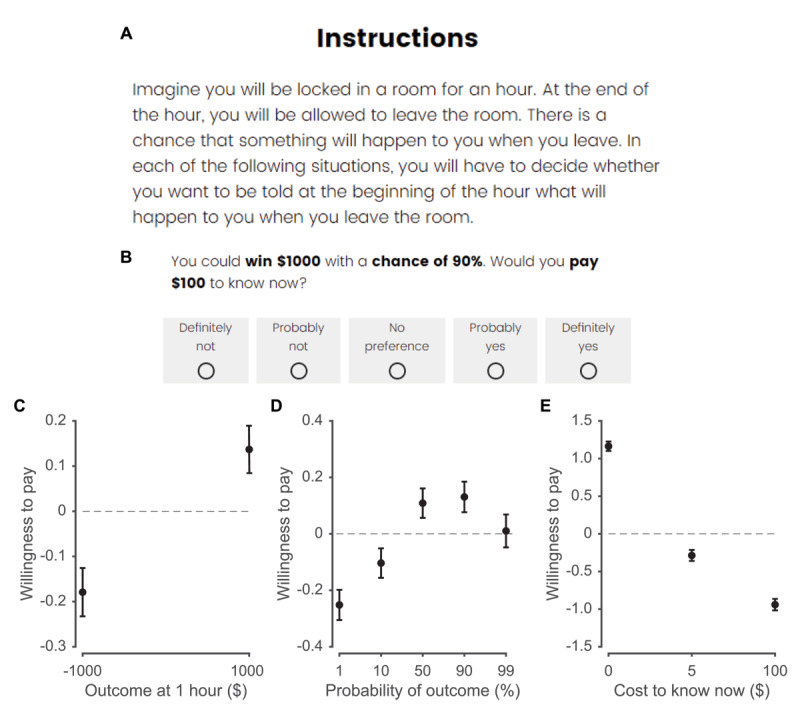
Task instructions and general findings. **A)** Task instructions. **B)** Example scenario. **C,D,E)** Average willingness to pay as a function of outcome at 1 hour (C), probability (D), and cost to know now (E) for the *N* = 227 subjects across 30 scenarios each. Willingness to pay is the Likert response for each scenario, scored from –2 for ‘definitely not’ to +2 for ‘definitely yes.’

Subjects read the following prompt (Figure 1A):

Imagine you will be locked in a room for an hour. At the end of the hour, you will be allowed to leave the room. There is a chance that something will happen to you when you leave. In each of the following situations, you will have to decide whether you want to be told at the beginning of the hour what will happen to you when you leave the room.

and were then given the following example scenario (Figure 1B):

You could win $1000 with a chance of 90%. Would you pay $100 to know now?

They responded on a Likert scale with one of the following answers: definitely not, probably not, no preference, probably yes, definitely yes. They then completed 30 scenarios in random order that differed by outcome (lose $1000, win $1000), probability of outcome at 1 hour (1%, 10%, 50%, 90%, 99%), and the cost to know now ($0, $5, $100). They were explicitly provided this information and instructed that their answers would not affect their payout.

### Anxiety inventories and factors

After finishing the task, subjects completed two anxiety inventories: the State-Trait Anxiety Inventory-Trait version (STAIT; Spielberger, 1983) and the State-Trait Inventory of Cognitive and Somatic Anxiety-Trait version (STICSAT; Grös et al., 2007).

The STAIT consists of 20 questions, each with 4 possible responses: almost never, sometimes, often, and almost always (Table S1). The STICSAT consists of 21 questions, each with 4 possible responses: not at all, a little, moderately, very much so (Table S2). Individual items were coded from 1 to 4, where 1 corresponded to no anxiety and 4 corresponded to high anxiety. For the anxiety-present questions on the STAIT (questions 2, 4, 5, 8, 9, 11, 12, 15, 17, 18, 20) and for all questions on the STICSAT, 1 corresponded to ‘almost never’/‘not at all’ responses and 4 corresponded to ‘almost always’/‘very much so’ responses. Anxiety-absent questions on the STAIT (questions 1, 3, 6, 7, 10, 13, 14, 16, 19) were reverse coded so a score of 1 corresponded to ‘almost always’ responses and a score of 4 corresponded to ‘almost never’ responses.

Following Fan et al. (2023), we used responses on the STAIT and STICSAT (41 total responses) and calculated scores on four factors for each subject. These oblique factors were identified on the basis of exploratory factor analysis and confirmatory factor analysis in an independent sample. Briefly, exploratory factor analysis identified 4 factors on the basis of parallel analysis. Confirmatory factor analysis, using four indices to compare quality of fit (comparative fit index, Tucker-Lewis Index, standardized-root-mean-square residual, and root-mean-square error of approximation) all favored 4 oblique factors. These factors were labeled somatic anxiety, cognitive anxiety, negative affect, and low self-esteem on the basis of item loadings. We refer the interested reader to Fan et al. (2023) for details. We used the factor loading structure from Fan et al. (2023) and calculated factor scores with the Bartlett method (Bartlett, 1937) using the psych package (version 2.4.3) in R (version 4.2.2).

### Resource-rational model of planning

We adapted the resource-rational model from Pierson and Goodman (2014). This model is based on the premise that people have limited cognitive resources and therefore seek information to determine whether they should devote resources to planning for outcomes. If an event is going to happen, then time and resources can be devoted to planning for it. If the event is not going to happen, then no resources need to be wasted planning for it. The agent is assumed to have three choices:

1. Obtain information: To obtain information, the agent pays a utility (C_info_) which we assume scales linearly with the cost to know now, *c*. If the outcome is going to occur, with probability *p*, the agent gains a utility associated with planning for the outcome (U_outcome_) minus a planning cost (C_plan_). To account for gain/loss asymmetries (Kahneman and Tversky, 1979), we scale U_outcome_ by s_gain_ when the outcome is positive and s_loss_ when the outcome is negative. If the outcome does not occurs, with probability 1–*p*, the agent does not pay a planning cost and gains utility of 0.
1 \[
{{\mathrm{U}}_{{\mathrm{info}}}} = p\left({{\mathrm{s}} \cdot {{\mathrm{U}}_{{\mathrm{outcome}}}}-{{\mathrm{C}}_{{\mathrm{plan}}}}} \right)-{{\mathrm{C}}_{{\mathrm{info}}}} \cdot c
a\]
  where U_info_ is the net utility associated with obtaining information.
2. Plan in uncertainty: The agent can choose to plan without paying for information. In this case, the agent always pays a planning cost (C_plan_) and gains a utility associated with the outcome (U_outcome_) with probability *p*, scaled by s_gain_ or s_loss_.
2 \[
{{\mathrm{U}}_{{\mathrm{plan}}}} = p \cdot {\mathrm{s}} \cdot {{\mathrm{U}}_{{\mathrm{outcome}}}}-{{\mathrm{C}}_{{\mathrm{plan}}}}
\]
  where U_plan_ is the net utility associated with planning in uncertainty.
3. Live in denial: Finally, the agent can choose neither to obtain information nor to plan for the outcome. We assume this frees up computational resources and the agent gains utility U_leisure_.

The agent makes a choice in accordance with the value of information, VOI, which is the difference between the utility of paying for information (U_info_) and the utility of not paying for information (the better of U_plan_ and U_leisure_; see Figure 3 for an intuition):

3 \[
{\mathrm{VOI}} = {{\mathrm{U}}_{{\mathrm{info}}}}-{\mathrm{max}}\left({{{\mathrm{U}}_{{\mathrm{plan}}}},{{\mathrm{U}}_{{\mathrm{leisure}}}}} \right)
\]

We constructed hierarchical models to estimate each parameter. For U_outcome_, C_plan_, C_info_, and U_leisure_, parameters were drawn from 𝒩(*μ*, 1) (parameterized as mean and standard deviation), where *μ* ∼ 𝒩(0, 5) for each parameter. The scale parameters, s_gain_ and s_loss_, were each drawn from 𝒩(1,1). All parameters were constrained to be greater than 0 and were drawn separately for each subject. To map the value of information onto Likert responses, we used an ordered logistic regression with 4 cut points corresponding to the 5 Likert categories. Given the apparent linear relationship between cost and responses, cost was rank transformed and recentered ([$0 $5 $100] → [0 1 2]) prior to model fitting. This simplification is generally consistent with concave utility functions (Pratt, 1978) used to model risk aversion.

Our model is similar to the model in Pierson and Goodman (2014) and simpler in several regards. In the original paper, subjects can devote computational resources *E* ∈ [0, 1] to planning for an event and gain utility *G(E)* if the event occurs, where *G(E)* is a monotonically increasing function. We simplified this by replacing *G(E)* and *E* each with a with a constant, U_outcome_ and C_plan_ respectively. As stated above, we remapped the cost to know now, implicitly implementing a concave utility function of risk aversion. Note that “Model 2: Risk aversion” in Pierson and Goodman (2014) parameterized this function, while we hold it fixed across all subjects. Our last inclusion was a version of “Model 3: Loss aversion.” We, instead, found that subjects were gain seeking in our task, and we therefore parameterized scaling parameters to capture this tendency.

We fit the models with R (version 4.2.2; accessed with RStudio 2022.12.0+353) using the Rstan package (version 2.26.13). We used 500 warmup samples and 1000 iterations over 4 chains, for a total of 2000 posterior samples per parameter. We fit the model with the default No-U-Turn Sampler algorithm. We constrained parameters to be greater than 0 by using the “lower=0” syntax (e.g., defining the utility of outcome variable as real<lower=0> U_outcome to impose a lower bound of 0). We otherwise used default settings. The ‘full’ model estimated 6 parameters per subject (U_outcome_, C_plan_, C_info_, U_leisure_, s_gain_ and s_loss_), 4 hyperparameters per subject (*μ* for U_outcome_, C_plan_, C_info_, and U_leisure_), 3 trial-by-trial variables per subject per trial (VOI, U_info_, U_plan_), and 4 cut points (to categorize responses into the 5 Likert categories).

We compared the model presented above to two reduced models: one with no outcome scaling (s_gain_ = s_loss_ = 1) and one with a single shared C_info_ parameter across all subjects. We performed model comparison using Pareto-smoothed importance sampling leave-one-out cross-validation to estimate the expected log predictive density, an established technique for Bayesian model comparison (Vehtari et al., 2017). We performed model comparison using the loo package (version 2.5.1). Our ‘full’ model above was strongly favored (Table S3). This model was identified based on our discovery sample and fixed for our pre-registered sample to limit analytic degrees of freedom.

**Figure 2:**
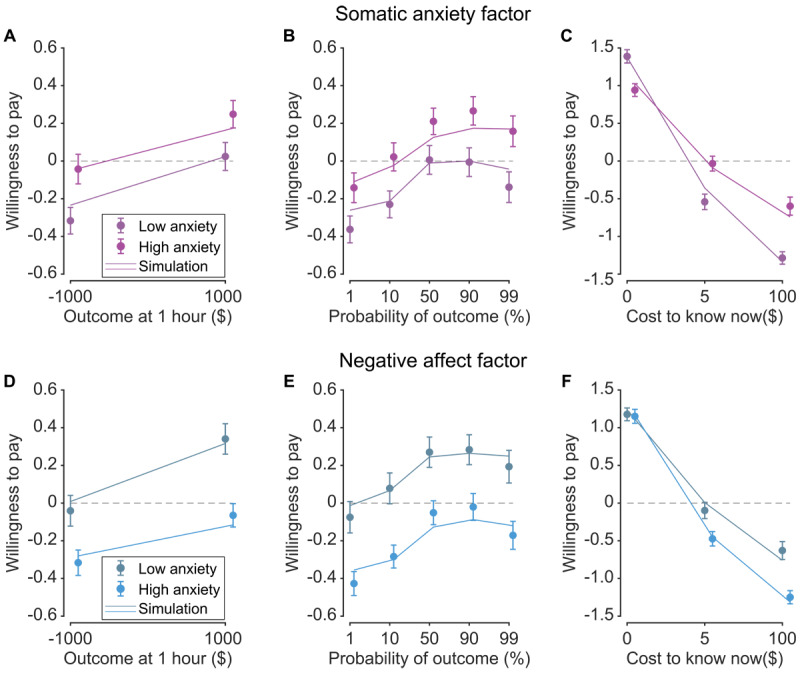
Willingness to pay as a function of somatic anxiety and negative affect. **A,B,C)** For somatic anxiety, willingness to pay as a function of outcome at 1 hour (A), probability of outcome (B), and cost to know now (C). **D,E,F)** For negative affect, willingness to pay as a function of outcome at 1 hour (D), probability of outcome (E), and cost to know now (F). Solid lines model simulations. For visualization only, data are median split into high anxiety (bright colors) and low anxiety (dull colors).

We performed posterior predictive checks by using the mean of the posterior distribution of parameter values of each subject to generate a full, synthetic dataset on the exact task performed by subjects. These simulations are presented in solid lines in Figures 1, 2, S1, and S4.

To ensure parameter identifiability, we used the synthetic dataset above and fit the ‘full’ model again. We computed Pearson’s correlations between the ground-truth and recovered parameters (with bootstrapped 95% confidence intervals), which revealed good recoverability of all parameters in the model (Table S4).

### Statistical analyses

For purposes of visualization, Likert responses were integer scored from –2 (for ‘definitely not’) to +2 (for ‘definitely yes’). To explore the relationship between task parameters and anxiety factor scores (without a process model), we fit a mixed-effects ordered logistic model predicting response (one of the 5 ordered Likert categories) with fixed effects of outcome at 1 hour, probability, cost to know now (and their interactions), and each of the four anxiety factor scores, and a random intercept per subject. Each input was *z*-scored prior to model fitting. We used the ordinal package (version 2023.12-4) in R (version 4.2.2). To determine the relationship between anxiety factor scores and model variables/parameters, we took the mean variable/parameter estimate for each participant and fit linear regressions predicting model variables/parameters as a function of the four anxiety factor scores. We used the fitlm function in MATLAB R2024a and standardized model inputs and outputs by *z*-scoring. We computed a single outcome scaling parameter by taking the log ratio of the scaling parameters: log(s_gain_∕s_loss_). Error bars are standard errors of the mean unless stated otherwise.

### Data availability

Data and code are available at https://osf.io/6zyxg/.

## Results

We replicated prior findings (Pierson and Goodman, 2014), finding that subjects generally desired to learn the outcome, even though it had no objective, instrumental value (Figure 1C). Across outcomes, subjects wanted to know more when the outcome was positive (*β* = 0.237, *p* < 10^–15^), when the probability was high (*β* = 0.170, *p* < 10^–11^), and when the cost was low (*β* = –1.324, *p* < 10^–15^). The relationship between willingness to pay and probability of outcome was flatter for losses than for gains (interaction between outcome and probability, *β* = 0.157, *p* < 10^–9^; Figure S1D-G, S2D-G).

Based on the intolerance of uncertainty account of anxiety, we hypothesized that subjects with high trait anxiety would have a greater willingness to pay for information, since access to this information reduces uncertainty. After performing the experiment, subjects completed two validated trait anxiety scales, the STAIT and STICSAT. On the basis of prior work, we extracted four anxiety factor scores for each participant: somatic anxiety, cognitive anxiety, negative affect, and low self-esteem (Fan et al., 2023). These labels were chosen on the basis of item loadings (Tables S1, S2). For each factor, the item with the highest loading was as follows: for somatic anxiety, ‘My face feels hot’; for cognitive anxiety, ‘I can’t get some thought out of my mind’; for negative affect, ‘I am content’; for low self-esteem ‘I lack self-confidence.’ In Figure 2 (Figure S7 for discovery sample), we highlight somatic anxiety and negative affect, as these two factors were the least correlated (Figure S3).

Consistent with our hypothesis, we identified a significant effect of somatic anxiety on willingness to pay, such that those with high anxiety were more willing to pay (*β* = 0.287, *p* = 0.0112; Figures 2A–C, S5A–C). This was evident as an increased willingness to pay regardless of outcome, probability, and cost to know (when nonzero), an insight we will build on further when modeling the cognitive processes underlying these decisions. The increased willingness to pay was specific to somatic anxiety; we did not identify a significant effect of cognitive anxiety (*β* = 0.107, *p* = 0.413; Figures S4A–C, S5D–F), or low self-esteem (*β* = 0.0860, *p* = 0.333; Figure S4D–F, S5J–L). Intriguingly, we identified a significant *decrease* in willingness to pay in those with high negative affect scores (*β* = –0.417, *p* < 10^–6^; Figure 2D–F, S5G–I). How do somatic anxiety and negative affect differentially modulate the desire for information?

**Figure 3:**
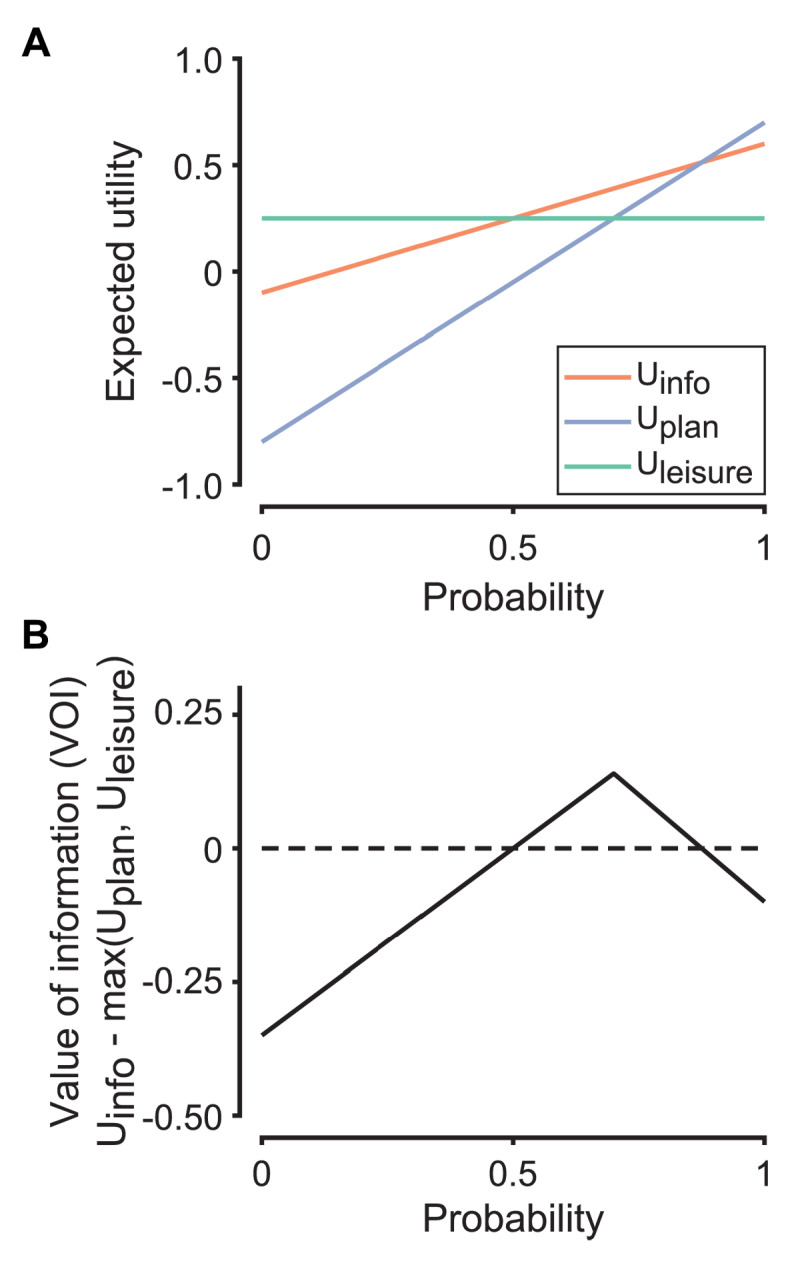
Example calculation of the value of information. **A)** Expected utilities of information (U_info_), planning in uncertainty (U_plan_), and living in denial (U_leisure_) as a function of probability of the outcome. For this simulation, we used s = 1, U_outcome_ = 1.5, C_plan_ = 0.8, C_info_ = 0.1, *c* = 1, and U_leisure_ = 0.25. **B)** The value of information as a function of probability. Here, there is an inverted-U relationship between the value of information and the probability of the outcome.

To gain insight into the underlying cognitive computations, we adapted a resource-rational model of planning developed by Pierson and Goodman (2014). Central to this model is the idea that brains are resource-limited and planning is costly: it makes sense to pay for apparently useless information if one wants to use that information to plan for future outcomes. Framed this way, people behave as if they are using the information for planning. The model distinguishes 3 different choices for each scenario: subjects can pay for information (and then decide whether they should plan), they can plan in uncertainty (i.e., commit to planning but without paying to know the outcome), or they can live in denial (i.e., neither planning nor paying for information). The key decision variable in this model is the value of information—the difference between the utility that can be gained by paying for information and the utility that can be gained by ignoring information (Figure 3). The model contains several parameters that govern the utility of the outcome, the cost of planning, the cost of information, the utility of leisure, and scaling of gains vs losses. We fit the model and found that it captured key features of behavior, including variation across anxiety factors (Figures 1, 2, S1, S4, and S6). We adhered closely to the modeling framework of Pierson and Goodman (2014) to remove analytical degrees of freedom. The general goodness of fit is a good sign that the analytical approach is robust, reinforcing the model’s explanatory power.

**Figure 4:**
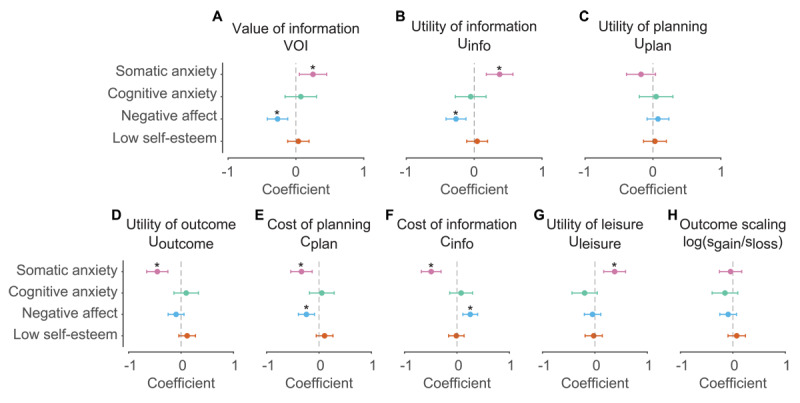
Relationship between anxiety factor scores and cognitive model variables and parameters. **A,B,C)** Standardized regression coefficients between anxiety factors and model variables. The model variables listed here are the value of information (A), the utility of information (B), and utility of planning (C). **D,E,F,G,H)** Standardized regression coefficients between anxiety factors and model parameters. The model parameters listed here are the utility of outcome (D), cost of planning (E), cost of information (F), utility of leisure (G), and the outcome scaling, represented as the log of the ratio between gains and losses (H). Error bars are 95% confidence intervals. * denotes *p* < 0.05.

We next constructed linear regression models to identify the association between anxiety factor scores and the variables/parameters in the cognitive model (Figure 4; Figure S8 for discovery sample). Consistent with our behavioral data, there was a significant increase in the value of information for the somatic anxiety factor (*β* = 0.252, *p* = 0.0144) and a decrease for the negative affect factor (*β* = –0.271, *p* = 4.86 × 10^–4^). For somatic anxiety, the increase in value of information arose from increased utility of information, the utility associated with paying for information (*β* = 0.375, *p* = 2.13 × 10^–4^). For negative affect, we identified the opposite; the decrease in value of information arose from decreased utility of information (*β* = –0.267, *p* = 4.24 × 10^–4^). We identified no significant changes in the utility of planning.

To gain insight into how the value of information was modulated by somatic anxiety and negative affect, we next regressed anxiety factor scores onto the model parameters. For somatic anxiety, we identified a fairly complex set of parameter changes. Somatic anxiety was associated with decreased utility of outcome (*β* = –0.453, *p* = 1.60 × 10^–5^), decreased cost of planning (*β* = –0.335, *p* = 1.42 × 10^–3^), decreased cost of information (*β* = –0.492, *p* = 5.65 × 10^–7^), and increased utility of leisure (*β* = 0.377, *p* = 4.61 × 10^–4^). This combinatorial change in parameters captures a curious feature of the data—the crossing over in willingness to pay as a function of cost to know now (Figure 2C). Negative affect was associated with decreased cost of planning (*β* = –0.240, *p* = 2.25 × 10^–3^) and increased cost of information (*β* = 0.254, *p* = 4.83 × 10^–4^). This combination decreases the utility of information, which in turn decreases the value of information.

## Discussion

We studied the relationship between trait anxiety and the desire for non-instrumental information, finding that subjects with high trait somatic anxiety consistently exhibited a stronger desire for non-instrumental information and that those with high negative affect exhibited a decreased desire for this same information. Using a resource-rational model of planning, we found that the utility of information was increased in somatic anxiety and decreased in negative affect.

Prior work has found marked heterogeneity in preferences for non-instrumental information (Bennett et al., 2016). Our work shows that some of this heterogeneity reflects previously unmeasured structure due to trait anxiety. A key strength of our work is in teasing apart multiple facets of anxiety. When we analyzed desire for information as a function of STAIT scores, a common approach for studying how anxiety contributes to cognitive processes, we found no relationship between anxiety and preferences for non-instrumental information (data not shown). Our findings only emerged after we built on prior work recognizing the multifactorial nature of anxiety and decomposed anxiety into multiple factors (Fan et al., 2023). This suggests that previous studies identifying no relationship between anxiety and information seeking may in fact be capturing the mixed influence of factors associated with increased and decreased information seeking (e.g., Charpentier et al., 2022).

The somatic anxiety factor appears quite related to clinical anxiety disorders. Generalized anxiety disorder, for example, requires at least 3 of the 6 following symptoms: restlessness, fatigue, difficulty concentrating, irritability, muscle tension, sleep disturbance. As such, our findings for somatic anxiety likely hold some insight into the behavior of high-anxiety people more generally. For example, an anxious student with an upcoming exam might endlessly ask questions of their professor to reduce uncertainty, or an anxious patient might ask numerous questions about potential medication side effects before deciding whether they want to take the medication. Clinically, somatic and cognitive anxiety are so highly correlated (Figure S3) that it is not typical in clinical practice to attempt to disambiguate the two to understand how much of a patient’s distress can be attributed to each symptom cluster. This is a clear strength of our approach, as clinical intuition alone would be insufficient to link somatic anxiety and not cognitive anxiety with information seeking behavior. The high correlation between these symptoms is reflected in diagnostic criteria, as can be seen for generalized anxiety disorder above where there is a mix of both physical and cognitive symptoms.

In contrast, the negative affect factor appears much less related to clinical anxiety; none of the symptoms of generalized anxiety disorder are consistent with sadness, low mood, etc… In the clinical setting, symptoms of negative affect are more easily disambiguated from the more classic anxiety symptoms of the somatic and cognitive anxiety factors. This is also suggested by the relatively weak correlation between negative affect and somatic/cognitive anxiety. In support of the independence of negative affect from anxiety, prior work has demonstrated that the items comprising the negative affect factor map more closely to depression (Bieling et al., 1998). Framed this way, the reduced desire for information is consistent with a depressive phenotype, typified by a more inwardly drawn experience and relative inattention to the outside word. While negative affect is likely distinct from a more “pure” anxiety construct, we hesitate to conflate it with depression since geriatric depression has been termed “depression without sadness,” suggesting depression and negative affect may also be independent from one another (Gallo and Rabins, 1999).

At first blush, one would think cognitive anxiety, not somatic anxiety, should be the key factor driving changes in information seeking, since changes in the process of thinking itself should engender changes in decision making. Our observation that trait somatic anxiety, not trait cognitive anxiety, correlates with increased preference for information is a strength of our approach, since it shows that this approach can allow us to draw seemingly counter-intuitive conclusions. We do not yet have a good foundational understanding of why somatic anxiety should be linked with changes in information preference. Note also that some previous studies have found no evidence for a relationship between somatic anxiety and information preferences (Witte et al., 2024), or even effects in the opposite direction (Fan et al., 2023). We discuss the latter finding more below.

Our study fails to provide evidence for the loss aversion account of anxiety, which states that anxiety arises from overestimating the consequences of negative events (Butler and Mathews, 1983; Hartley and Phelps, 2012; Paulus and Angela, 2012). If loss aversion were the primary driver of trait anxiety, we would have captured it as a stronger preference towards negative outcomes and a change in the loss scaling parameter. Our findings generally favor intolerance of uncertainty, consistent with recent work suggesting the same (Charpentier et al. 2017; though see Charpentier et al. 2022).

Our support for the intolerance of uncertainty hypothesis is a bit more nuanced than it might appear. One point of contention is the recent finding that somatic anxiety is associated with *decreased* exploration of uncertain options (directed exploration) in a bandit task (Fan et al., 2023) which appears at odds with our findings of *increased* information-seeking in a non-instrumental task. We offer two explanations for why this may be the case. First, when cost is zero, somatic anxiety is associated with *decreased* desire for information (Figure 2C). In the bandit task of Fan et al. (2023), information has a zero monetary cost; perhaps the lack of financial disincentive led to decreased directed exploration. Our findings suggest that introducing financial costs for information may lead to *increased* directed exploration. Second, Bennett et al. (2020) offers another potential resolution. In a non-instrumental task, these authors found that subjects with high anxiety had increased preference for costly information *and*, independently, decreased preference for high-variance outcomes. The aversion towards high-variance outcomes may therefore be consistent with decreased directed exploration in Fan et al. (2023). Although this reconciles our disparate findings, it suggests nuance to the intolerance of uncertainty hypothesis which would predict increased directed exploration in anxiety. Separately, the fact that our findings of increased information-seeking in anxiety is consistent with Bennett et al. (2020) argues for the robustness of this finding.

The concordance between our findings and those of Bennett et al. (2020) is important for a second reason. Our study used hypothetical scenarios without feedback while Bennett et al. (2020) had subjects make choices that resulted in real feedback. The behavioral differences under these two scenarios has been summarized under the so-called “hypothetical bias” (Harrison and Rutström, 2008; List and Gallet, 2001; Murphy et al., 2005). For example, in hypothetical scenarios, participants tend to overstate their willingness to pay or show more risk-tolerant behaviors. Similar findings have been made under the stated vs revealed preferences research program (Manski, 1990; Carson et al., 1996). The convergence of our findings with Bennett et al. (2020) suggests our observations are insensitive to low level experimental details.

Under our framework, the relationship between outcome variance and anxiety could be studied by parameterizing a concave utility cost function, like those used to model risk aversion (Pratt, 1978). In fact, Pierson and Goodman (2014) developed such a model (their Model 2), though we opted not to implement it for simplicity. We implicitly used a concave utility function in remapping the cost to know now from [$0 $5 $100] to [0 1 2], but we did not parameterize it (it was the same mapping for all participants). This therefore precluded our ability to investigate the relationship between trait anxiety and loss aversion. Studying how anxiety and loss aversion relate to one another would likely require task manipulations in which outcome variance is changed independently of outcome magnitude.

In summary, by applying a resource-rational model of planning, we find that trait somatic anxiety correlates with an increased desire for information and that trait negative affect correlates with a decreased desire for information. Intriguingly, we identified a number of changes in trait somatic anxiety related to the allocation of cognitive resources (U_outcome_, C_plan_, U_leisure_). Although interpreting each parameter through a clinical lens is challenging, the net sum may explain the fatigue and poor concentration that are common to anxiety disorders (e.g., Bishop, 2009; Hallion et al., 2018)—these subjects engage in planning more often and for subjectively devalued outcomes, taxing cognitive resources. This effect may be further compounded by inefficient planning (Unterrainer et al., 2018). The relationships we have identified here just scratch the surface and the connection between trait anxiety and planning is a rich area for future work.

## Additional File

The additional file for this article can be found as follows:

10.5334/cpsy.124.s1 Supplemental Information. Figures S1 to S8 and Tables S1 to S4.
