# Supplementary material for: The Value of Non-Instrumental Information in Anxiety: Insights from a Resource-Rational Model of Planning
Source: Comput Psychiatr. 2025 Feb 12;9(1):63–75. doi: 10.5334/cpsy.124 (PMC11827562; doi:10.5334/cpsy.124)
Supplement: Supplemental Information. — Figures S1 to S8 and Tables S1 to S4. [file cpsy-9-1-124-s1.pdf]

Supplemental Information

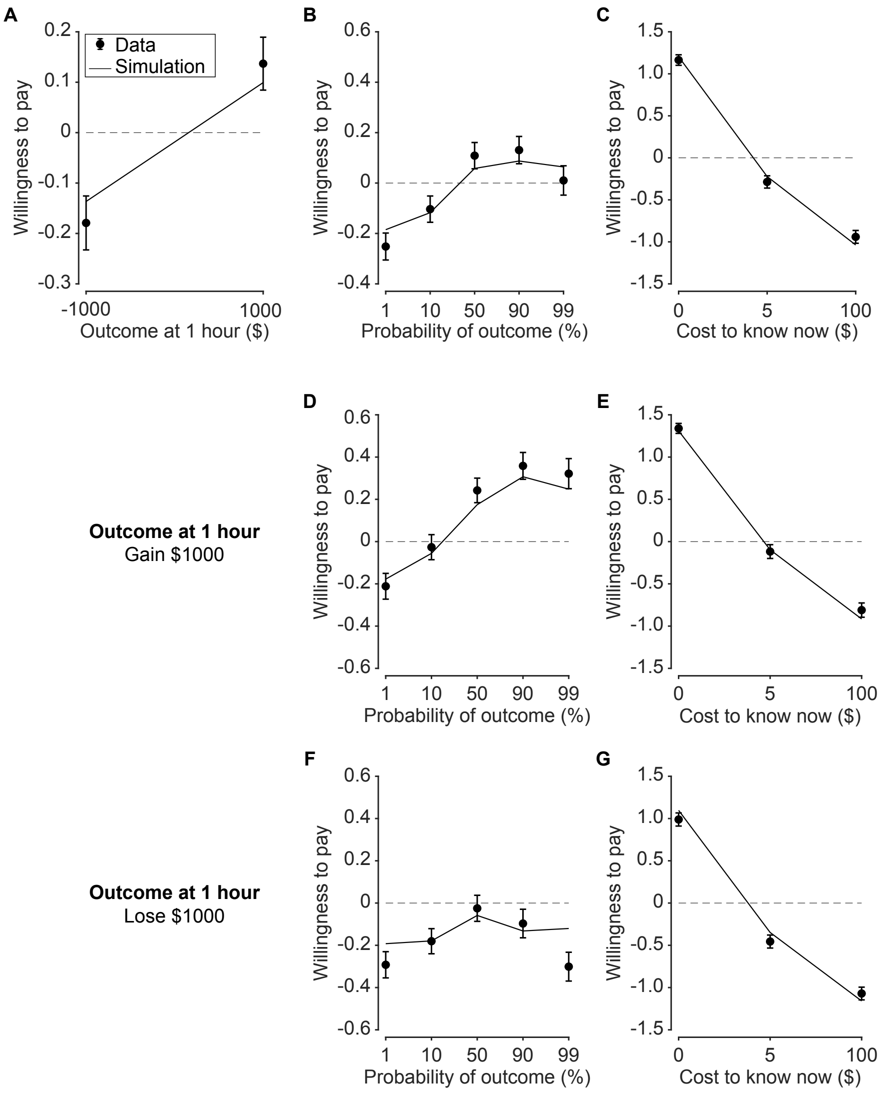

Figure S1: Willingness to pay, separately for gains and losses, with model simulations. A,B,C) Willingness to pay as a function of outcome at 1 hour (A), probability (B), and cost to know now (C), overlaid with model simulations (solid lines). The behavioral data are the same as in Figure 1C-E.

D,E) Willingness to pay for scenarios where the outcome was positive.

F,G) Willingness to pay for scenarios where the outcome was negative.

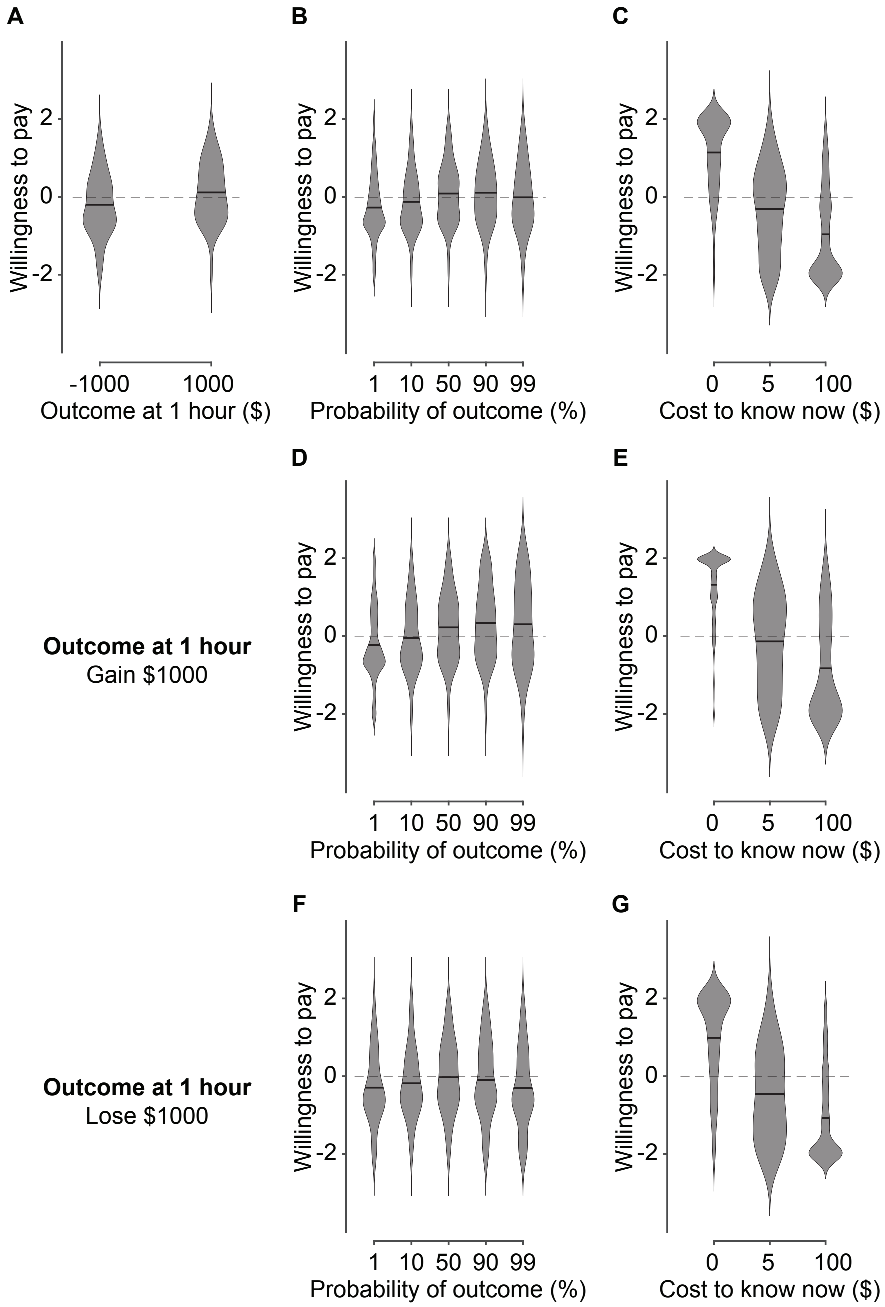

Figure S2: Violin plots of willingness to pay, separately for gains and losses.
A,B,C) Willingness to pay as a function of outcome at 1 hour (A), probability (B), and cost to know now (C).
D,E) Willingness to pay for scenarios where the outcome was positive.
F,G) Willingness to pay for scenarios where the outcome was negative.
The data are the same as in Figures 1C-E and S1. Black line is the mean.

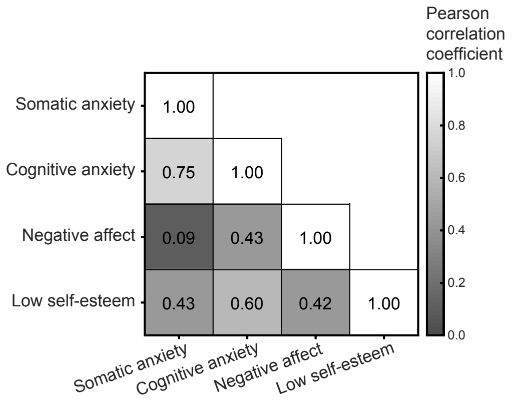

Figure S3: Anxiety factor scores correlation matrix.

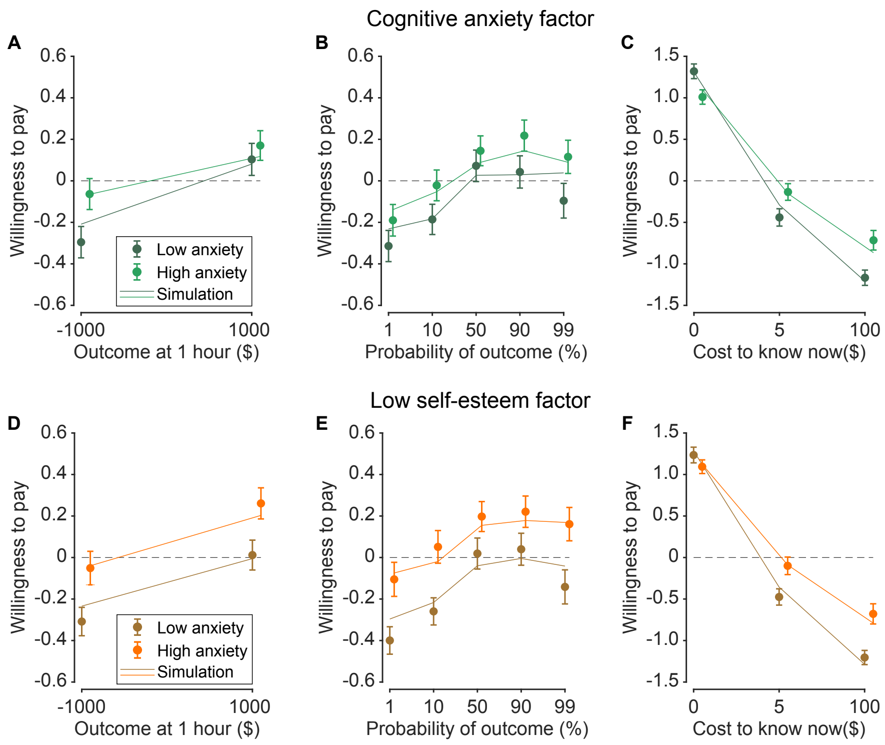

Figure S4: Willingness to pay as a function of cognitive anxiety and low self-esteem. A,B,C) For cognitive anxiety, willingness to pay as a function of outcome at 1 hour (A), probability of outcome (B), and cost to know now (C).
D,E,F) For low self-esteem, willingness to pay as a function of outcome at 1 hour (D), probability of outcome (E), and cost to know now (F).
The apparent differences in willingness to pay as a function of cognitive anxiety and low self-esteem are due to correlations between anxiety factors (Figure S3) and disappear when all factors are considered simultaneously (Figure 4).

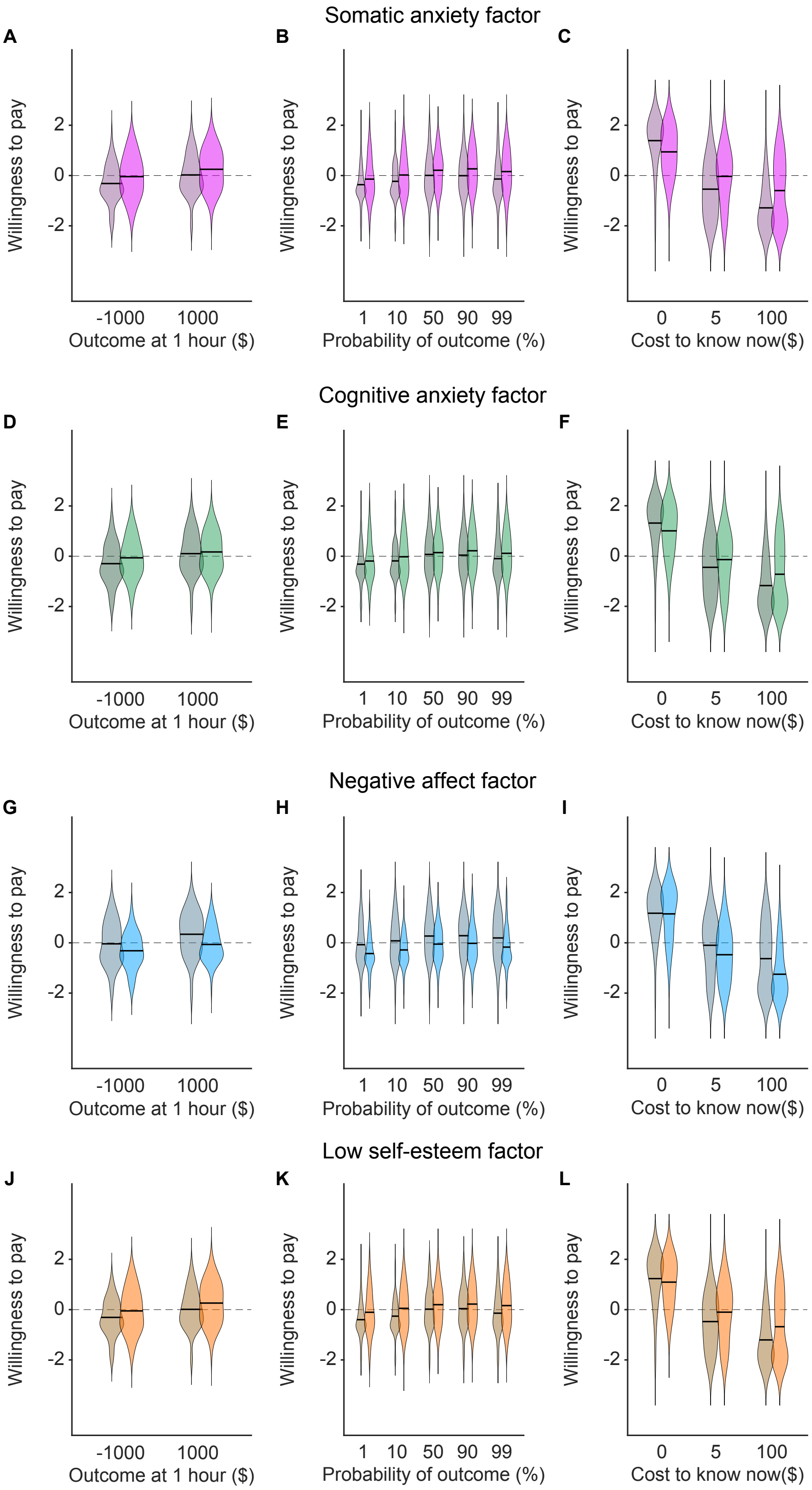

Figure S5: Violin plots of willingness to pay as a function of anxiety factor scores.
A,B,C) For somatic anxiety, willingness to pay as a function of outcome at 1 hour (A), probability of outcome (B), and cost to know now (C).
D,E,F) The same for cognitive anxiety.
G,H,I) The same for negative affect.
J,K,L) The same for low self-esteem.
The data are the same as in Figures 2 and S4. Black line is the mean.

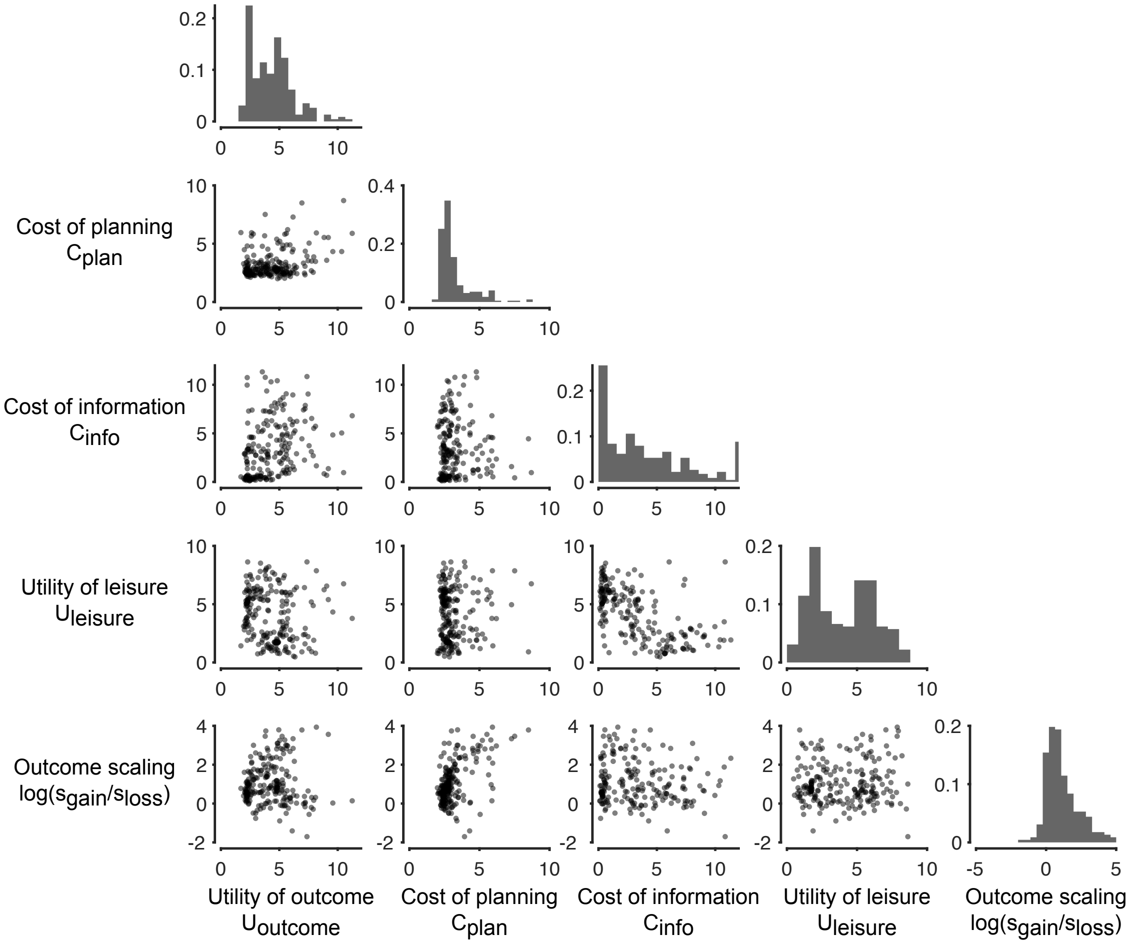

Figure S6: Parameter estimates for each participant.
The histograms on the diagonal show the marginal distribution of parameter estimates across all participants, for each of the 5 model parameters, where the y-axis is the probability. The point estimate was taken as the the mean of each subject's posterior parameter distribution. The off-diagonal scatter plots show pairwise parameter estimates for each parameter combination.

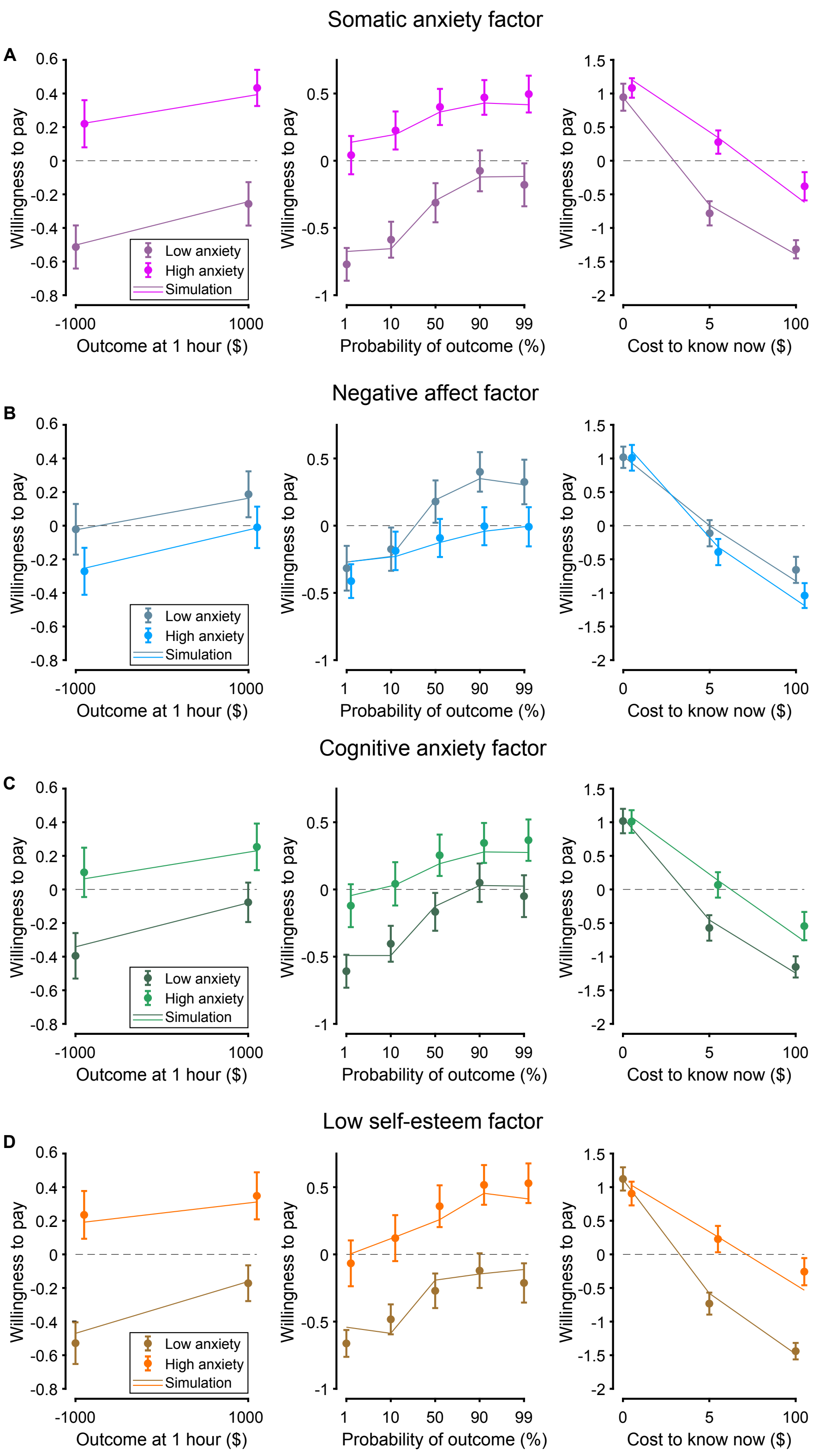

Figure S7: Discovery sample: Relationship between anxiety factor scores and willingness to pay.
A) For somatic anxiety, willingness to pay as a function of (from left to right) outcome at 1 hour, probability of outcome, and cost to know now. B) Same for negative affect. C) Same for cognitive anxiety. D) Same for low self-esteem. The relationship between somatic anxiety and willingness to pay was significant in the discovery sample ($\beta = 0.895$, $p = 3.15 \times 10^{-4}$).
The apparent differences in willingness to pay as a function of cognitive anxiety and low self-esteem are due to correlations between anxiety factors and disappear when all factors are considered simultaneously.

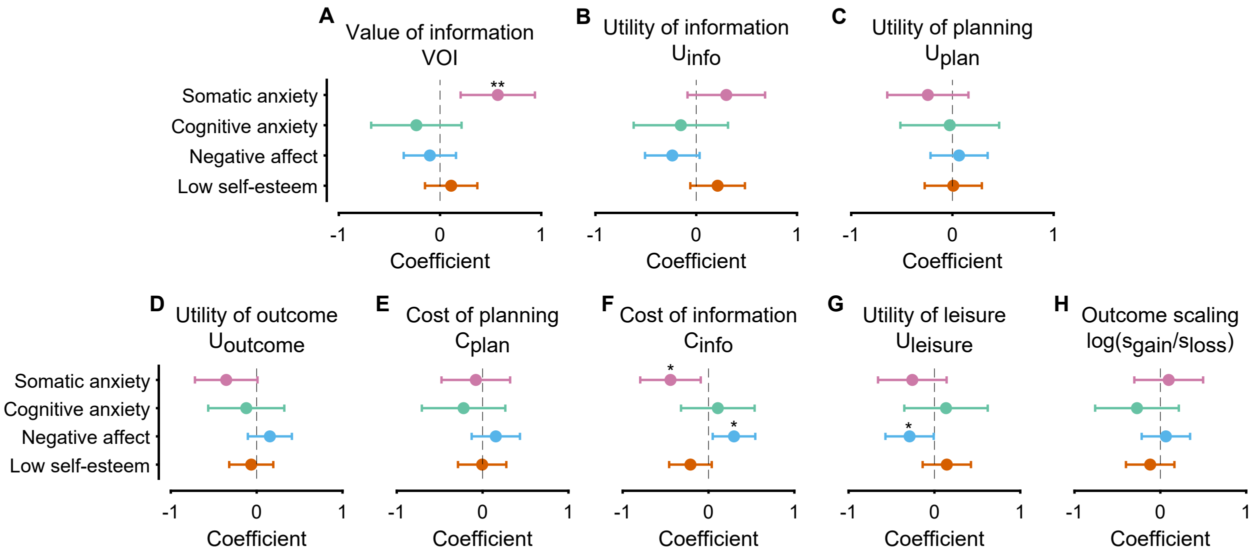

Figure S8: Discovery sample: Relationship between anxiety factor scores and cognitive model variables.

A,B,C) Standardized regression coefficients between anxiety factors and model variables. The model variables listed here are the value of information (A), the utility of information (B), and utility of planning (C).

D,E,F,G,H) Standardized regression coefficients between anxiety factors and model parameters. The model parameters listed here are the utility of outcome (D), cost of planning (E), cost of information (F), utility of leisure (G), and the outcome scaling, represented as the log of the ratio between gains and losses (H).

Error bars are 95% confidence intervals. * denotes $p < 0.05$.

STAIT Item	Anxiety factor loadings			
	Somatic anxiety	Cognitive anxiety	Negative affect	Low self-esteem
1.* I feel pleasant	-0.019	0.115	**0.811**	-0.064
2. I feel nervous and restless	**0.301**	**0.356**	0.217	0.089
3.* I feel satisfied with myself	-0.128	0.099	**0.720**	0.171
4. I wish I could be as happy as others seem to be	0.058	0.182	0.106	**0.322**
5. I feel like a failure	0.100	0.292	0.179	**0.441**
6.* I feel rested	-0.007	-0.030	**0.722**	-0.087
7.* I am 'cool, calm, and collected'	-0.002	0.077	**0.797**	-0.062
8. I feel that the difficulties are piling up so that I cannot overcome them	0.297	0.229	0.176	**0.335**
9. I worry too much over something that doesn't really matter	0.031	**0.558**	0.087	0.169
10.* I am happy	-0.017	-0.000	**0.823**	0.059
11. I have disturbing thoughts	0.248	**0.393**	-0.006	0.269
12. I lack self-confidence	0.149	0.129	0.143	**0.521**
13.* I feel secure	0.053	-0.058	**0.825**	0.072
14.* I make decisions easily	-0.123	0.213	**0.673**	-0.090
15. I feel inadequate	0.101	0.264	0.186	**0.497**
16.* I am content	0.075	-0.157	**0.846**	0.054
17. Some unimportant thoughts run through my mind and bother me	-0.102	**0.785**	-0.075	0.169
18. I take disappointments so keenly that I can't put them out of my mind	-0.081	**0.655**	-0.025	0.228
19.* I am a steady person	-0.004	-0.072	**0.773**	0.030
20. I get in a state of tension or turmoil as I think over recent concerns and interests	0.080	**0.513**	0.019	0.265

Table S1: Factor loadings on the STAIT.
The anxiety-absent items marked by * were reverse-scored prior to computing factor weights.

STICSAT Item	Anxiety factor loadings			
	**Somatic anxiety**	**Cognitive anxiety**	**Negative affect**	**Low self-esteem**
1. My heart beats fast	**0.741**	-0.019	0.077	0.008
2. My muscles are tense	**0.718**	0.094	0.135	-0.062
3. I feel agonized over my problems	0.120	**0.676**	0.110	0.063
4. I think that others won't approve of me	0.165	**0.524**	0.068	0.180
5. I feel like I'm missing out on things because I can't make up my mind soon enough	0.124	**0.672**	-0.004	0.046
6. I feel dizzy	**0.807**	0.001	-0.007	-0.025
7. My muscles feel weak	**0.757**	0.087	0.074	-0.053
8. I feel trembly and shaky	**0.775**	0.100	0.030	-0.039
9. I picture some future misfortune	0.194	**0.582**	0.104	0.075
10. I can't get some thought out of my mind	-0.060	**0.918**	0.040	-0.072
11. I have trouble remembering things	**0.383**	**0.425**	0.075	-0.061
12. My face feels hot	**0.861**	-0.056	-0.143	0.078
13. I think that the worst will happen	0.229	**0.546**	0.182	0.062
14. My arms and legs feel stiff	**0.831**	-0.010	0.043	0.011
15. My throat feels dry	**0.724**	0.062	0.017	0.061
16. I keep busy to avoid uncomfortable thoughts	0.048	**0.755**	-0.054	-0.036
17. I cannot concentrate without irrelevant thoughts intruding	0.096	**0.824**	0.014	-0.069
18. My breathing is fast and shallow	**0.861**	-0.032	-0.042	0.043
19. I worry that I cannot control my thoughts as well as I would like to	0.141	**0.747**	0.043	-0.032
20. I have butterflies in the stomach	**0.737**	0.059	-0.066	0.021
21. My palms feel clammy	**0.722**	0.038	-0.131	0.037

Table S2: Factor loadings on the STICSAT.

Model	Expected log predictive density difference ± SE
Full model	Reference
No outcome scaling ($s_{gain} = s_{loss} = 1$)	-122.6 ± 23.4
Shared C_{info} across all subjects	-741.2 ± 33.7

Table S3: Model comparison using Pareto-smoothed importance sampling leave-one out cross validation. A difference in the expected log predictive density of 4 points provides evidence in favor of a model. The 'Full model' is strongly favored over the others. The 'No outcome scaling' model weights gains and losses equally ($s_{gain} = s_{loss} = 1$). The 'Shared C_{info}' model has a single C_{info} shared across all subjects.

Parameter	Pearson correlation between ground-truth and recovered parameter (95% CI)
$U_{outcome}$	0.818 (0.710 - 0.884)
C_{plan}	0.728 (0.460 - 0.872)
C_{info}	0.984 (0.969 - 0.991)
$U_{leisure}$	0.959 (0.932 - 0.976)
s_{gain}	0.716 (0.538 - 0.817)
s_{loss}	0.824 (0.721 - 0.891)

Table S4: Parameter recovery. The 'Full model' was fit to all subjects to generate a ground-truth set of parameter estimates. Mean parameter estimates for each subject were used to generate a synthetic dataset using the exact same task that the subjects completed. The 'Full model' was fit to this synthetic dataset to generate a recovered set of parameters. We estimated the mean parameter estimates for each subject and computed the Pearson correlation between the ground-truth parameter estimates and the recovered parameter estimates. We computed 95% confidence intervals by bootstrap sampling.
